# Role of the N-terminal lid in regulating the interaction of phosphorylated MDMX with p53

**DOI:** 10.18632/oncotarget.22829

**Published:** 2017-12-01

**Authors:** Jane Vin Chan, Dawn Xin Ping Koh, Yun Liu, Thomas L. Joseph, David P. Lane, Chandra S. Verma, Yaw Sing Tan

**Affiliations:** ^1^ Bioinformatics Institute, Agency for Science, Technology and Research (A*STAR), Singapore; ^2^ p53 Laboratory, Agency for Science, Technology and Research (A*STAR), Singapore; ^3^ Department of Biological Sciences, National University of Singapore, Singapore; ^4^ School of Biological Sciences, Nanyang Technological University, Singapore

**Keywords:** MDMX/MDM4, phosphotyrosine, p53, protein–protein interaction, molecular dynamics

## Abstract

Murine double minute 4 protein (MDMX) is crucial for the regulation of the tumor suppressor protein p53. Phosphorylation of the N-terminal domain of MDMX is thought to affect its binding with the transactivation domain of p53, thus playing a role in p53 regulation. In this study, the effects of MDMX phosphorylation on the binding of p53 were investigated using molecular dynamics simulations. It is shown that in addition to the previously proposed mechanism in which phosphorylated Y99 of MDMX inhibits p53 binding through steric clash with P27 of p53, the N-terminal lid of MDMX also appears to play an important role in regulating the phosphorylation-dependent interactions between MDMX and p53. In the proposed mechanism, phosphorylated Y99 aids in pulling the lid into the p53-binding pocket, thus inhibiting the binding between MDMX and p53. Rebinding of p53 appears to be facilitated by the subsequent phosphorylation of Y55, which draws the lid away from the binding pocket by electrostatic attraction of the lid's positively charged N-terminus. The ability to target these mechanisms for the proper regulation of p53 could have important implications for understanding cancer biology and for drug development.

## INTRODUCTION

The p53 tumor suppressor protein is crucial in protecting our body from diseases such as cancer [1]. When DNA damage occurs, p53 acts as a transcription factor to activate its target genes, resulting in cell cycle arrest and apoptosis. It is normally kept at low levels due to tight regulation by two major inhibitors, mouse double minute 2 and 4 homologs (MDM2 and MDMX respectively, also referred to as HDM2 and HDMX in humans), which bind to the transactivation domain of p53 [2] to reduce p53 activity. During cellular stress, release of the p53 protein from the MDM proteins is required for p53 activation. However, following cell repair, the MDM proteins are required to rebind p53 to bring p53 activity back to normal levels.

The MDM proteins are homologs that contain two distinct conserved domains, an N-terminal p53-binding domain and a C-terminal RING domain, separated by an extended and largely disordered central region of more than 300 residues. Previous studies have focused on the structural basis of the MDM2–p53 interaction and the mechanism by which this interaction is regulated [3–5]. However, either MDM2 or MDMX alone is insufficient to effectively inhibit p53 due to their functional dependence on each other. MDMX is required to stabilize MDM2 and prolong its half-life, thereby allowing it to target p53 for degradation, while the nuclear localization sequence of MDM2 is required to direct MDMX into the nucleus to carry out functional inhibition of p53 [6]. These functions are mediated by the interaction of the C-terminal RING domains of MDM2 and MDMX to form a MDM2/MDMX heterocomplex that is essential for the E3 ubiquitin ligase activity of MDM2. Disruption of this heterocomplex has been shown to result in marked p53 activation [7]. Thus, an understanding of the mechanism and regulation of the MDMX–p53 interaction is just as important as that of the MDM2–p53 interaction.

**Figure 1:**
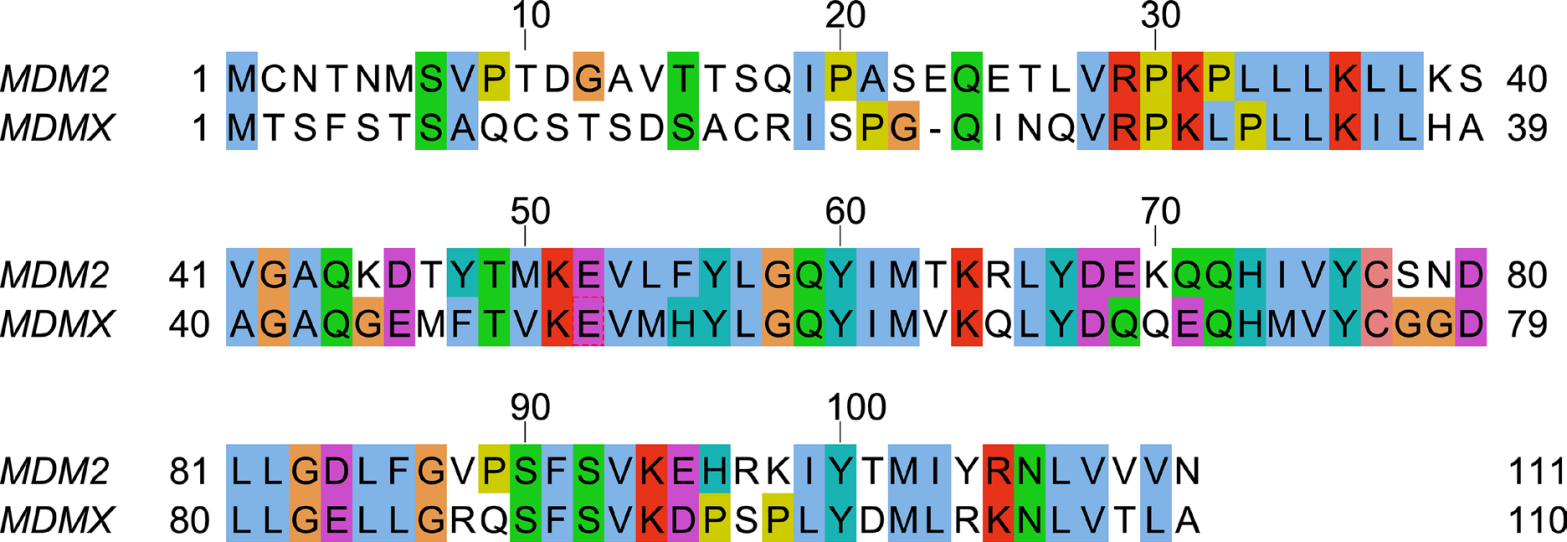
Sequence alignment of the N-terminal domains of human MDM2 and human MDMX The figure was generated by Jalview [51].

MDM2 and MDMX bind to p53 at their N-terminal domains to inhibit its transactivation. However, unlike MDM2, MDMX is unable to target p53 for proteolysis or induce its nuclear export [8]. Instead, MDMX remains localized in the cytoplasm in the absence of MDM2 [9]. Within their N-terminal p53-binding domains, MDMX and MDM2 share an amino acid residue identity of 53.6% (Figure 1) [10]. A hydrophobic surface groove is present at the p53-binding site of both MDM2 and MDMX, allowing for three key hydrophobic residues from p53 (F19, W23, and L26) to bind to the MDM proteins [11].

**Figure 2:**
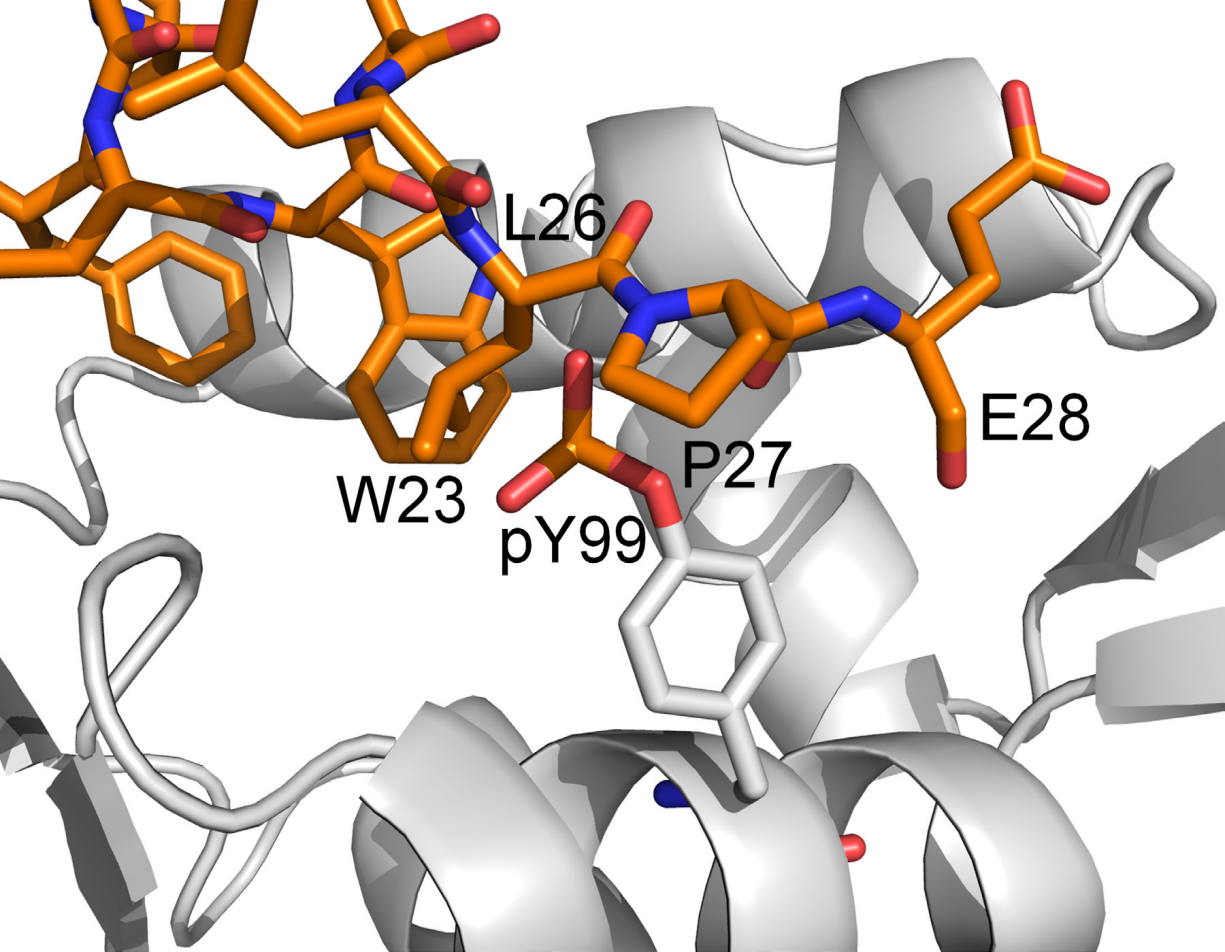
Model (generated from the PDB structure 3DAB) proposed by Zuckerman et al [14] to explain the inhibitory effect of Y99 phosphorylation on p53 binding to MDMX. The phosphotyrosine is shown to clash sterically with P27 of p53.

A significant body of literature exists on how stress-activated signals result in the activation of p53 by phosphorylation of MDM2 and p53, and the consequential release of p53 from MDM2 [12]. However, there is a paucity of such information for MDMX [13]. This inspired a study by Zuckerman *et al.* [14], in which they studied the site-specific phosphorylation of the N-terminal domain of MDMX by the tyrosine kinase c-Abl. It was reported that phosphorylation of Y99 in MDMX impairs the interaction between MDMX and p53. This was proposed to be due to the introduction of the charged phosphate group of phosphorylated Y99 (pY99) into the hydrophobic p53-binding pocket, causing steric clash with P27 of p53 (Figure 2) and resulting in the release of p53 from MDMX. Although this is an unlikely conformation for pY99 to adopt due to the high desolvation penalty involved, a recently released crystal structure of MDMX-pY99 (MDMX with Y99 phosphorylated) in complex with a high-affinity MDMX-binding peptide called PMI appears to support this model [15]. Due to the presence of the bulky phosphate group of pY99 near the p53-binding site, the C-terminus of PMI is displaced from the binding pocket. A lateral shift of the entire peptide from its original position in MDMX also occurs. However, the authors also note that the steric clash with P27 does not entirely account for the deleterious effect of Y99 phosphorylation and that other structural factors may be involved.

**Figure 3:**
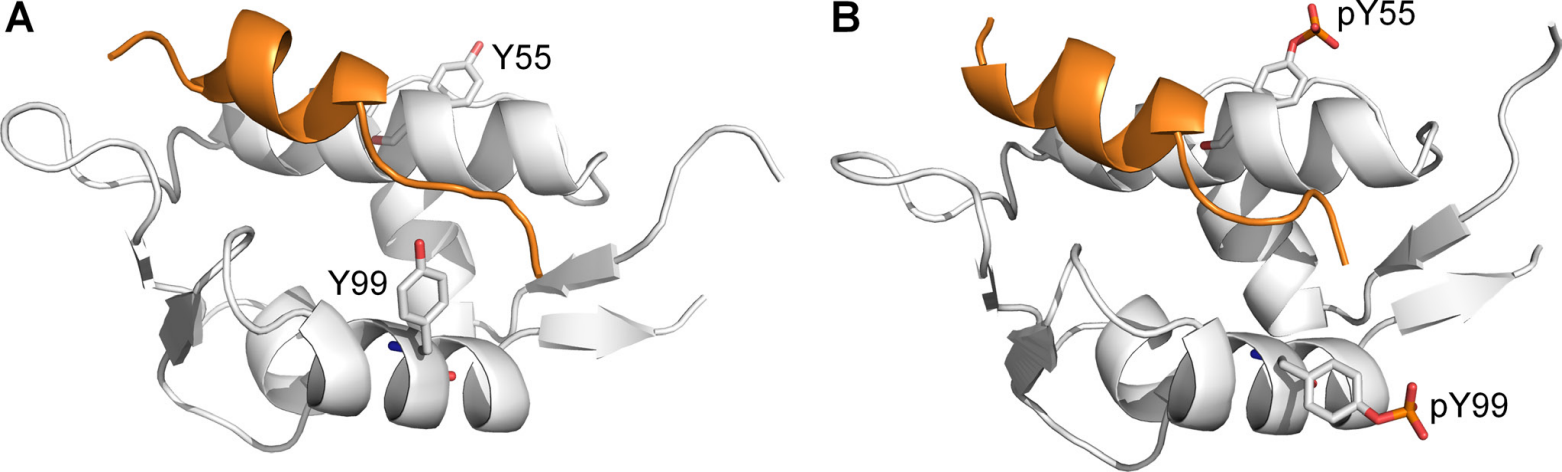
Structures of p53 peptide (orange) in complex with (**A**) nonphosphorylated MDMX with Y99 in the ‘closed’ conformation (PDB 3DAB), and (**B**) diphosphorylated MDMX (white) with pY99 in the ‘open’ conformation (structure obtained from MD simulation).

Within the same study by Zuckerman *et al.* [14], it was also discovered that phosphorylation of Y55 follows Y99 phosphorylation, resulting in an enhanced MDMX–p53 interaction when both Y99 and Y55 are phosphorylated. Due to the time-dependent phosphorylation of these tyrosine residues, the enhanced MDMX–p53 interaction observed after Y55 phosphorylation is likely to be due to p53 rebinding to MDMX, which helps in the downregulation of p53 following stress-induced activation. However, Y55 is located away from the p53-binding site (Figure 3A), and therefore unable to directly affect the interaction with p53. The mechanism by which phosphorylated Y55 (pY55) enhances the binding between MDMX and p53 is unclear. Considering that Y99 is still phosphorylated and blocking the p53 binding site, the doubly phosphorylated MDMX should not be able to rebind p53. Hence, it is unlikely that steric clash between pY99 and p53 is the sole reason for the latter's release from MDMX. It is possible that other factors are involved in the interaction of phosphorylated MDMX with p53 and they are missing from the current model.

Although both Y55 and Y99 are conserved in MDM2 and MDMX, there is currently no evidence to show that these residues are phosphorylated in MDM2. Instead, in a previous study on the roles of post-translational modifications of MDM2 in the regulation of the MDM2–p53 interaction [4], it was proposed that simultaneous phosphorylation of S17 on the intrinsically disordered N-terminal lid of MDM2 (residues 1*–*24), and T18 and S20 of p53 brings negatively charged residues from both molecules into close proximity, resulting in the disruption of the MDM2–p53 interaction. In the absence of p53, the MDM2 lid remains in close contact with the p53-binding site of MDM2 (the ‘closed state’), stabilizing MDM2 by shielding the hydrophobic site. It adopts a highly flexible ‘open state’ in p53-bound MDM2 [16]. Although the lid also competes weakly with p53, it is easily displaced by other MDM2 ligands. Since MDM2 and MDMX are structurally homologous, it is likely that the N-terminal region of MDMX, analogous to the MDM2 lid, engages in a similar interaction with the p53-binding site. However, the role and function of the MDMX N-terminal lid are not well understood, and hence, it is often neglected in structural and binding studies of MDMX. For example, the lidless version of MDMX (residues 24–108) was used for X-ray crystallography and binding assays in the recent study that reports the crystal structure of MDMX-pY99 bound to PMI [15]. In this study, we examined the possible role of the N-terminal lid of MDMX in regulating the interaction of phosphorylated MDMX with p53.

Molecular dynamics (MD) simulation is a widely used molecular modelling technique that has been used to understand and successfully predict protein dynamics and structures in numerous studies [17–20]. Here, we performed extensive MD simulations using models of MDMX with its N-terminal lid to investigate the molecular mechanism by which the interaction between p53 and phosphorylated MDMX is regulated. This could provide new insights for the design of highly effective and improved anticancer MDMX inhibitors whose potencies are independent of the phosphorylation state of MDMX.

## RESULTS

### Phosphorylated Y99 adopts an ‘open’ conformation

To determine the effects of phosphorylation on MDMX, MD simulations of apo MDMX and p53-bound MDMX were performed (simulation sets 1 and 2, Supplementary Table 1). The crystal structure of the N-terminal domain of MDMX (residues 23–109) in complex with p53 (residues 17–28) was used as the initial structure for the simulations (PDB accession code 3DAB) [21]. For each simulation set, three subsets of simulations with MDMX in various phosphorylated states were carried out: the first with no residues phosphorylated, the next with Y99 phosphorylated, and the last with both Y99 and Y55 phosphorylated.

When MDMX is ligand-bound, Y99 usually adopts the ‘closed’ conformation, in which it points towards the p53-binding cleft, but there is structural evidence to show that it can also adopt the ‘open’ conformation, in which it points away from the binding pocket [22]. It is not known how Y99 behaves in the absence of a bound ligand, as no structures of apo MDMX have been experimentally determined. The conformations of Y99 and pY99 during the simulations of apo MDMX and p53-bound MDMX were determined by measuring their χ1 side chain dihedral angles. Y99 and pY99 are defined as being in the closed conformation when χ_1_ is in the range of 250°–300° (Figure 3A), while their open conformations corresponded to χ_1_ values in the range of 150–200° (Figure 3B).

**Figure 4:**
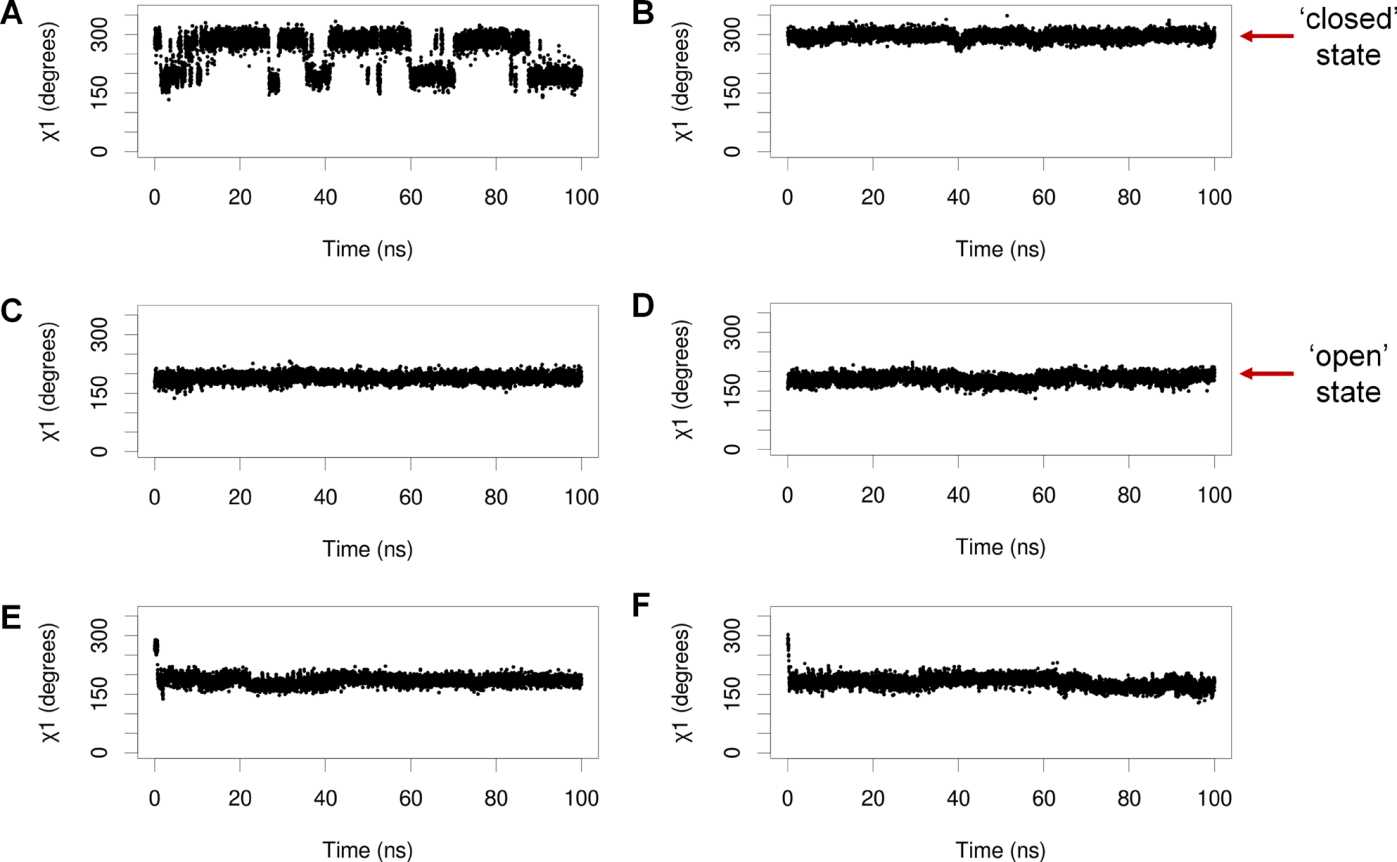
Representative plots of Y99/pY99 side chain dihedral angles (χ_1_) against time during simulations of (**A**) apo MDMX, (**B**) MDMX bound to p53, (**C**) apo MDMX-pY99, (**D**) MDMX-pY99 bound to p53, (**E**) apo MDMX-pY99-pY55, (**F**) MDMX-pY99-pY55 bound to p53.

Y99 fluctuated between the closed and open states in the simulations of apo MDMX (Figure 4A). In contrast, it could only adopt the closed state in the simulations of p53-bound MDMX (Figure 4B). This was due to the formation of a hydrogen bond between the hydroxyl group of Y99 and the carbonyl oxygen of P27 in the p53 peptide.

Upon phosphorylation in apo MDMX, pY99 remained in the closed state for a very short period before it flipped to the open state, where it remained for the rest of the simulations (Figure 4C). It was also consistently observed to adopt only the open conformation in the simulations of MDMX-pY99 bound to p53, apo MDMX-pY99-pY55 (MDMX with Y99 and Y55 phosphorylated), and MDMX-pY99-pY55 bound to p53 (Figure 4D–4F). The strict preference of pY99 for the open conformation is not entirely unexpected, as it is energetically unfavorable for its charged phosphate group to remain in close proximity with the hydrophobic p53-binding cleft. The open conformation not only allows the phosphate group to form favorable polar interactions with the bulk water molecules, but also relieves the steric clash with P27 of p53 predicted in the Zuckerman study.

The binding free energies of p53 in complex with the various phosphorylated states of MDMX were then compared using the molecular mechanics/generalized Born surface area (MM/GBSA) method [23]. For each system, the average binding free energy was determined from three independent MD runs. Based on the results from the Zuckerman study, Y99 phosphorylation would be expected to reduce the binding affinity of p53 with MDMX, while Y55 phosphorylation enhances it. However, the results of the energetic analysis show that the phosphorylation status of MDMX has little effect on the binding free energies of the MDMX–p53 complex (Table 1). Phosphorylation of Y99 did not significantly reduce the binding affinity of p53. Similarly, it remained relatively unchanged when Y55 was phosphorylated.

**Table 1:** Calculated binding free energies of p53 in complex with MDMX in different phosphorylation states

MD run	MDMX state	Average binding free energy (kcal/mol)
2.1	unphosphorylated	−7.7 ± 0.1
2.2	Y99 phosphorylated	−6.7 ± 2.4
2.3^a^	Y99 and Y55 phosphorylated	−7.1 ± 1.2

^a^Average binding free energy for run 2.3 was determined from two replicates, due to incomplete equilibration in one of the replicates.

The Zuckerman model assumes that pY99 does not deviate from its closed conformation and hence, will clash sterically with p53. However, our simulations of both apo and p53-bound MDMX-pY99 and MDMX-pY99-pY55 show that when pY99 is allowed to be flexible, it is able to rotate away from the p53-binding site and point towards the bulk solvent to relieve the steric clash. The binding energy calculations also indicate that this conformational change has little effect on the binding affinity of p53. Clearly, the mechanism by which pY99 weakens the binding of MDMX to p53 is not as simple as previously thought.

Notably, in both the Zuckerman model and the crystal structure of MDMX-pY99 bound to PMI (13), the N-terminal lid of MDMX is conspicuously absent. Likewise, the N-terminal lid was missing from the MDMX crystal structure that was used to initiate our simulations. It was similarly neglected in early structural and binding studies. However, recent studies have shown that the N-terminal lid of MDM2 is highly involved in the regulation of p53 binding to MDM2 (14, 15). The N-terminal lid of MDMX could also play an analogous role here. We incorporated it into our simulations to see if we could elucidate a role for it in the regulation of p53 binding to phosphorylated MDMX.

### Effects of phosphorylation on the behavior of N-terminal lid in apo MDMX

**Figure 5:**
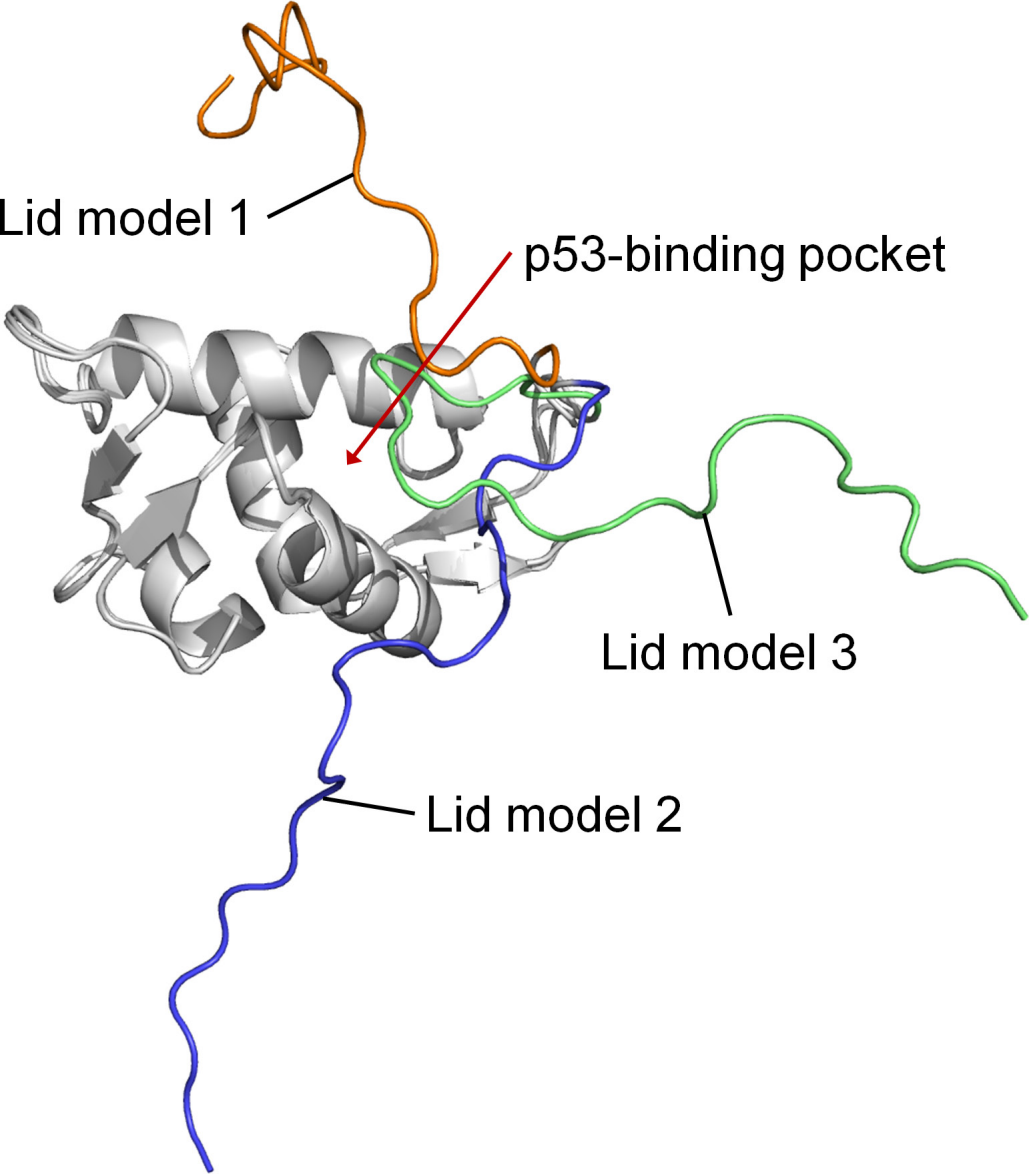
Superimposition of the three models of the N-terminal domain of MDMX with its lid

Secondary structure prediction software [24, 25] suggest that the N-terminal lid of MDMX is unstructured, like that of MDM2. Since the N-terminal lid (residues 1–22) is not resolved in any of the known MDMX structures, it was created by homology modeling based on the solution structure of the N-terminal domain of apo MDM2 [26]. The rest of the N-terminal domain of MDMX (residues 23–109) was derived from the crystal structure (PDB code 3DAB) that was used to initiate the MD simulations. Three representative models of the lid, each occupying a different conformational space relative to the p53-binding cleft, were generated (Figure 5). In lid model 1, the lid partially occludes the binding site, while in lid model 2, it lies almost perpendicular to the binding site. Lastly, lid model 3 has the lid pointing away from the binding site. These three structures represent a diverse set of initial MDMX lid conformations for the subsequent MD simulations.

MD simulations of these three models of MDMX in various phosphorylated states (nonphosphorylated, Y99 phosphorylated, Y99 and Y55 both phosphorylated) were performed (simulation sets 3–5, Supplementary Table 1). An additional set of simulations that mimics the process of sequential phosphorylation, in which Y99 is phosphorylated after 100 ns while Y55 is phosphorylated after 200 ns, was also carried out on each of the MDMX lid models.

The N-terminal lid showed a clear preference for occupying the p53-binding pocket in simulations of MDMX lid model 1. This was likely to be due to the initial position of the lid above the binding cleft, which predisposes the lid to collapse onto it. In contrast, the lid preferred to stay out of the binding cleft in lid models 2 and 3, due to the lid being further away from the binding site initially. Despite this, the lid ended up bound to the binding site in a few of these simulations. It was held within the binding cleft by multiple interactions involving mainly nonpolar residues of the lid (F4, A8, C10, A16, C17, I19) and the binding cleft.

The behavior of Y99 and pY99 in these simulations of MDMX with the lid is largely similar to that observed in the simulations of MDMX without its lid. Y99 oscillated between the closed and open conformations, while pY99 flipped to the open conformation where it remained. However, in 6 of the 27 simulations in which Y99 was phosphorylated, the lid was observed to pin down on pY99, forcing it to adopt the closed conformation. The lid did not occupy the p53-binding site in these simulations. In contrast, the lid was able to occupy the binding site with Y99 in the closed conformation in two simulations of unbound MDMX (lid models 1 and 2). These observations suggest a link between pY99 conformation and lid state. A closed pY99 conformation could prevent the lid from occupying the binding pocket by steric hindrance, forcing the lid to adopt the open state; while an open pY99 conformation exposes the binding pocket, allowing access to it by the lid, which can then adopt the closed state. No such correlation exists between Y99 conformation and lid state, as the lid was observed to bind to the binding pocket regardless of whether Y99 was in the open or closed conformation. This suggests a dual mechanism for the attenuated interaction between p53 and MDMX upon Y99 phosphorylation. The closed conformation of pY99, which is associated with the open state of the lid, results in steric clash with P27 of p53, while the open conformation of pY99, which is associated with the closed state of the lid, results in occupation of the binding site by the lid.

To better understand the molecular mechanism by which pY99 and pY55 regulate p53 binding to MDMX, the interactions of their side chains with other residues during the simulations were identified (Table 2). In particular, pY99 was often observed to interact with R103, which is one α-helix turn away. The formation of this salt bridge is one of the reasons pY99 prefers the open conformation in the simulations. pY99 also engages in another prominent salt bridge interaction with R18, which is located in the lid region. A few other lid residues form hydrogen bonds with pY99, but the pY99-R18 interaction has the highest occupancy. This particular interaction was observed in several MDMX-pY99 simulations where the lid was eventually found to be in the closed state. Taken together, these observations indicate an important role of the lid in MDMX–p53 binding. The phosphorylation of Y99 causes its side chain to adopt the open conformation, due to the unfavorable interaction with the hydrophobic p53-binding site and formation of a salt bridge with R103. This exposes the binding site for occupation by the lid. The open conformation of pY99 also allows it to form a salt bridge with R18 in the lid, which may play a role in initiating the movement of the lid towards the p53-binding site. As a result, the lid is now able to better compete for association with the p53-binding cleft, resulting in reduced binding of p53 to MDMX (12).

**Table 2:** Occupancies^a^ of hydrogen bonds formed by the side chain atoms of Y99, pY99 and pY55 in the last 100 ns of simulation sets 3–5

Acceptor	Donor (main/side chain)	Occupancy (%)		
		Nonphosphorylated MDMX	MDMX-pY99	MDMX-pY99-pY55
Y99	R18 (main)	9	ND^b^	ND
Y99	C10 (main)	8	ND	ND
S3	Y99 (side)	7	ND	ND
S96	Y99 (side)	6	ND	ND
pY99	R103 (side)	ND	33	36
pY99	R18 (side)	ND	22	19
pY99	S20 (side)	ND	ND	12
pY99	S5 (side)	ND	11	ND
pY99	T2 (side)	ND	9	11
pY99	S3 (side)	ND	9	ND
pY99	F4 (main)	ND	8	ND
pY55	H54 (side)	ND	ND	12
pY55	T2 (side)	ND	ND	12
pY55	S3 (side)	ND	ND	11
pY55	S5 (side)	ND	ND	11
pY55	R18 (side)	ND	ND	10
pY55	M1 (main)	ND	ND	5

^a^If a donor residue hydrogen bonds to pY99/55 via multiple donor hydrogens, only the highest occupancy is reported.

^b^ND indicates that the specified hydrogen bond was not detected.

pY55 also interacts with several residues in the lid region, most notably with the three N-terminal residues, M1, T2 and S3. These interactions were observed in trajectory structures that had the lid in the open state. It is likely that the interactions between pY55 and the N-terminal residues are responsible for drawing the lid away from the p53-binding site. The binding site is now exposed for p53 to approach and rebind, which could neutralize the inhibitory effect of pY99 on MDMX–p53 binding (12).

### Stable interaction between p53 and MDMX-pY99-pY55 with N-terminal lid

The simulations of the MDMX-pY99-pY55 lid models (sets 3–5, Supplementary Table 1) suggest that pY55 promotes the open state of the lid by direct interaction with its N-terminus. With the p53-binding cleft now exposed, MDMX-pY99-pY55 should be primed to rebind p53. To verify this model, another set of simulations in which p53 was reintroduced into MDMX-pY99-pY55 was carried out. From the final trajectory frames of the simulations of the MDMX-pY99-pY55 lid models (runs 3.3, 4.3, 5.3, Supplementary Table 1), three structures of MDMX-pY99-pY55 with the N-terminus of the lid in close proximity with pY55 and the lid in the open state (Supplementary Figure 1) were selected for the subsequent simulations (set 6, Supplementary Table 1).

p53 was observed to rebind in a stable conformation to two of the chosen MDMX-pY99-pY55 structures. This was reflected in their average binding free energies of −8.3 ± 1.2 kcal mol^-1^, which is comparable to that for the complex of unphosphorylated MDMX with p53 (Table 1). The binding free energy for the third run was not evaluated as p53 could not attain a stable bound conformation. This could be due to the absence of a direct and strong interaction between pY55 and N-terminus of the lid in the starting structure (Supplementary Figure 1C), which is necessary to maintain the lid in the open state. In contrast, pY55 engages in a favorable charge–charge interaction with the amino group at the N-terminus of the lid in the other two structures. This kept the lid clear of the p53-binding cleft, and facilitated the rebinding of p53 to MDMX.

These results suggest a possible mechanism for the rebinding of p53 to MDMX-pY99-pY55, in which pY55 induces an open lid state that exposes the binding pocket of MDMX for p53 to rebind. This would explain the enhancement of the MDMX–p53 interaction following Y55 phosphorylation, as observed by Zuckerman *et al.* in their study (12).

### N-terminal acetylation in MDMX inhibits interaction between N-terminal lid and pY55

**Figure 6:**
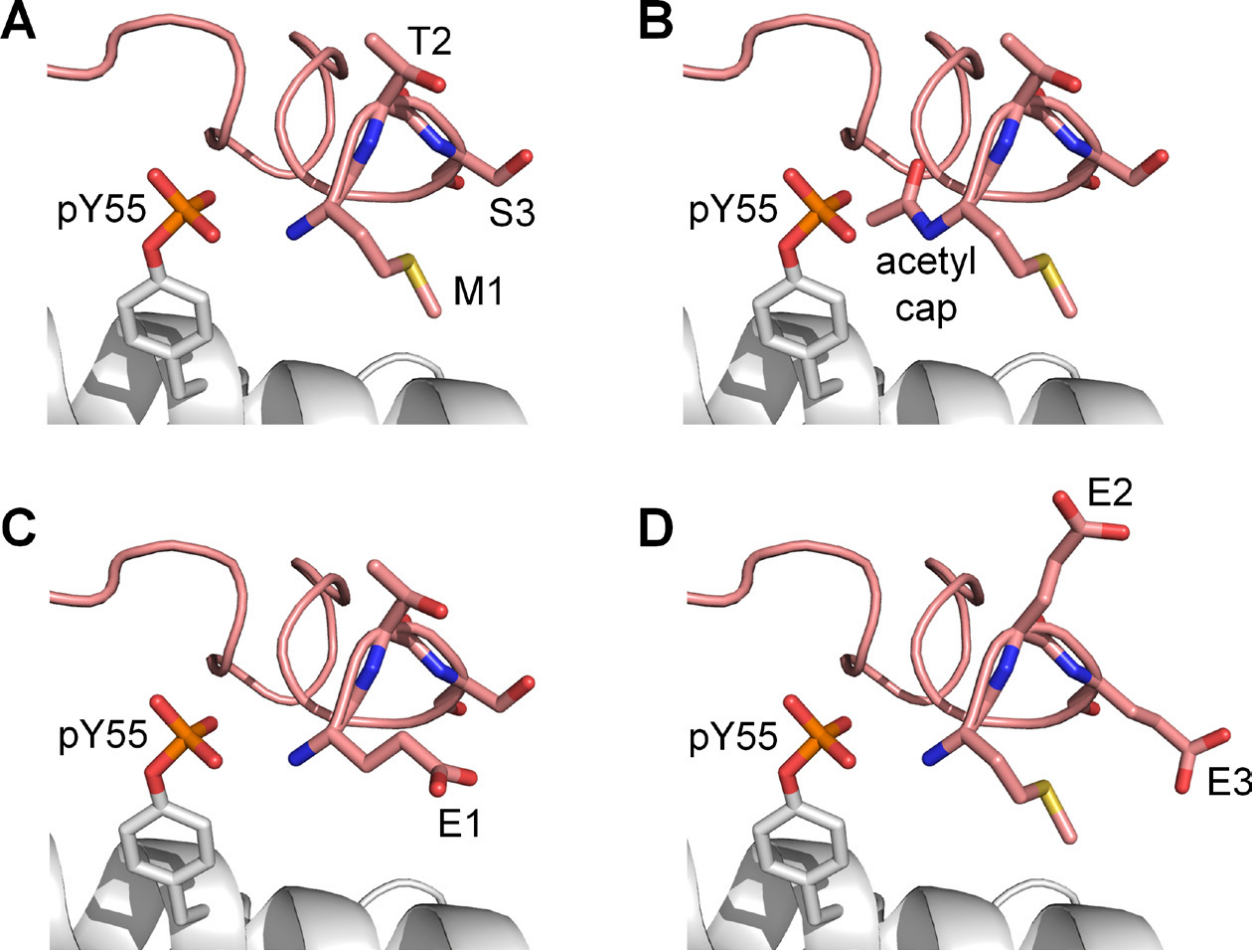
Modifications made to the N-terminal lid (pink) to weaken its interactions with pY55 (**A**) Unmodified lid. (**B**) N-terminal acetylation. (**C**) M1E mutation. (**D**) T2E and S3E mutations.

As the hydrogen bond interactions between the phosphate group of pY55 and the N-terminal residues were found to be important in keeping the lid in an ‘open’ state, several modifications to the N-terminus of the lid to abrogate its interactions with pY55 were modeled. This was done to identify modifications that could effectively interfere with the rebinding of p53 to MDMX-pY99-pY55 by allowing the N-terminal lid to return to the p53-binding site. Three different lid modifications were made to the same MDMX structures that were used for the previous set of p53 rebinding simulations (Supplementary Figure 1). The first modification involved the removal of the positive charge at the N-terminus of MDMX by acetyl capping (Figure 6B). The second modification was an M1E mutation (Figure 6C), while the third modification consisted of T2E and S3E double mutations (Figure 6D). It was hoped that the replacement of these N-terminal residues with glutamate would create a region of negative charge near the N-terminus of the lid that repels the phosphate group of pY55.

N-terminal acetylation proved to be the most effective at releasing the lid from pY55 during the simulations (sets 7–9, Supplementary Table 1). The lid moved away from pY55 in eight out of nine runs in which the N-terminus of MDMX was capped with an acetyl group (Table 3). When M1E or T2E and S3E double mutations were carried out, however, the lid remained close to pY55 in most runs. These results suggest that acetyl capping could be used to nullify the effect of Y55 phosphorylation by allowing the lid to move away from pY55, thus increasing its likelihood of returning to the p53-binding cleft to assume the closed state. Hence, N-terminally acetylated MDMX with both Y99 and Y55 phosphorylated is predicted to exhibit reduced affinity for p53 compared to unmodified MDMX.

**Table 3:** Effect of lid modifications on lid interaction with pY55

MD runs	Lid modification	No. of simulations	
		Lid moves away from pY55	Lid remains close to pY55
7.1, 8.1, 9.1	N-terminal acetylation	8	1
7.2, 8.2, 9.2	M1E	1	8
7.3, 8.3, 9.3	T2E, S3E	2	7

Three simulation runs were performed for each of the three modified MDMX lid models, for a total of nine runs per modification.

### R18E mutation in MDMX reduces interaction between N-terminal lid and pY99

To evaluate the importance of the interaction between pY99 and R18 for lid closing, four final trajectory structures of apo MDMX-pY99 (Supplementary Figure 2) with the pY99–R18 interaction and the lid in the binding pocket were selected for the next set of simulations (set 10, Supplementary Table 1). R18 was mutated to Glu in each structure. The negatively charged side chain of Glu was expected to repel the similarly charged phosphate group of pY99, thus abolishing the interaction between the two residues and facilitating the release of the lid from the binding cleft.

**Figure 7:**
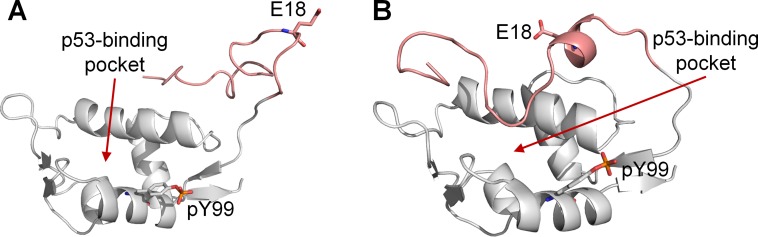
Final trajectory structures of MDMX-pY99-R18E (white) The N-terminal lid is shown in pink. (**A**) Final structure obtained from run 10.1, in which the lid was found to be completely out of the p53-binding pocket. (**B**) Final structure obtained run 10.3, in which the C-terminal segment of the lid was no longer at the p53-binding site.

Indeed, the C-terminal segment of the lid (residues 14–22), including E18, was no longer near the p53-binding site at the end of all the simulations (Figure 7). Several interactions between the p53-binding site and lid residues (A16, C17, I19) were also lost. These suggest that the electrostatic interaction between pY99 and R18 in MDMX could play a key role in inducing and maintaining the closed state of the lid. If this is indeed the case, binding assays should reveal an increase in binding affinity of p53 for MDMX-R18E-pY99 relative to MDMX-pY99, as the lid is unable to assume the closed state in the mutated form, leaving the binding cleft exposed for p53 binding.

### Glutamate as a phosphotyrosine mimetic

The production of phosphorylated proteins for experiments is difficult as the appropriate enzyme has to be used to perform the phosphorylation, and even then, it may be challenging to control which residue is being phosphorylated. Hence, aspartate and glutamate are routinely used as phosphomimetics to mimic the properties of phosphoserine and phosphothreonine. Although it is not common, glutamate has also been used as a natural amino acid mimetic of phosphotyrosine [27, 28]. We performed simulations to evaluate the suitability of glutamate as a phosphomimetic for phosphotyrosine in MDMX.

**Figure 8:**
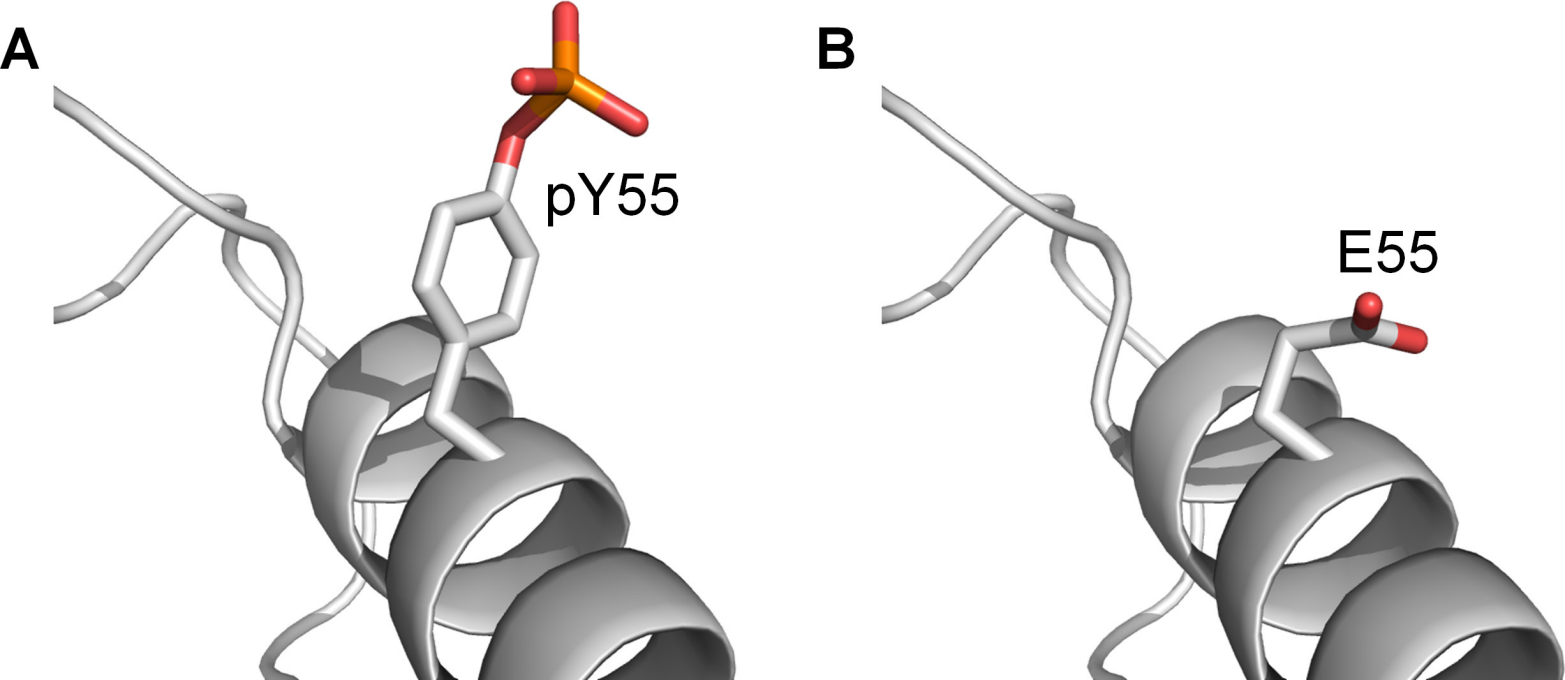
The structure of phosphotyrosine compared to glutamate (**A**) pY55 on MDMX-pY99-pY55. (**B**) E55 on MDMX-E99-E55.

For each of the three MDMX lid models, two mutated MDMX structures were generated: one carrying a Y99E mutation and the other carrying the Y99E and Y55E double mutations. These six structures were then subject to long MD simulations (sets 11–13, Supplementary Table 1). Table 4 shows a summary of the residues that interacted with E99 and E55 during the simulations. It was observed that E99 was able to interact with R18 in the lid, but the interaction was not as frequent and persistent as pY99. More importantly, E55 was unable to interact with the N-terminal residues of MDMX in all the simulations of MDMX-Y99E-Y55E (MDMX with Y99E and Y55E mutations). Assuming that our proposed model of the interaction of phosphorylated MDMX with p53 is correct, these simulations suggest that glutamate is not an effective phosphomimetic of pY55. Although both residues are negatively charged, glutamate has little chemical and structural similarity to phosphotyrosine [29] (Figure 8). Under physiological conditions, glutamate carries only a -1 charge, as compared to the -2 charge of phosphotyrosine. The side chain of glutamate is also much shorter than that of phosphotyrosine. These differences could explain the lack of interaction between E55 and the N-terminal residues of MDMX in the simulations. Our result agrees with experimental studies demonstrating the unsuitability of glutamate as a phosphotyrosine mimetic [29, 30].

**Table 4:** Occupancies^a^ of hydrogen bonds formed by the side chain atoms of E99 and E55 in the last 100 ns of simulation sets 11–13

Acceptor	Donor (main/side chain)	Occupancy (%)	
		MDMX-Y99E	MDMX-Y99E-Y55E
E99	R18 (side)	17	14
E99	R103 (side)	11	19
E99	S20 (main)	12	ND
E99	S7 (side)	ND	9
E99	S5 (side)	ND	9
E99	S20 (side)	8	6
E99	S13 (side)	5	6
E99	C17 (main)	5	ND
E55	R18 (side)	ND	13
E55	Q58 (side)	ND	6

^a^If a donor residue hydrogen bonds to E99/55 via multiple donor hydrogens, only the highest occupancy is reported.

## DISCUSSION

MDMX is an inhibitor of p53, and the mechanism by which MDMX releases and rebinds p53 is important for understanding p53 regulation in cancer biology and therapy. Phosphorylation of Y99 on MDMX has been found to impair MDMX–p53 interaction, supposedly due to steric hindrance caused by the phosphate group, while phosphorylation of Y55 on MDMX enhances MDMX–p53 interaction by an unknown mechanism. However, since Y55 phosphorylation has been shown to preferentially occur after Y99 phosphorylation, the MDMX–p53 interaction should remain impaired when both tyrosines are phosphorylated. The current proposed model is therefore still incomplete as the steric clash caused by pY99 only partially explains the attenuated interaction of MDMX and p53 following Y99 phosphorylation.

In this study, MD simulations were carried out to provide insight into the molecular mechanism by which phosphorylated MDMX releases and rebinds p53. Our results suggest that steric clash between p53 and pY99 of MDMX is not the only reason for impaired MDMX–p53 interaction upon Y99 phosphorylation. Instead, the N-terminal lid of MDMX is also likely to play an important role in the binding of p53 to MDMX, regulated by the phosphorylation of tyrosine residues. Y99 is phosphorylated under cellular stress. This is proposed to increase the interactions of the residues in the lid, particularly R18, with the phosphate group of pY99, which adopts the open conformation. This brings the lid close to the p53-binding site, thus facilitating the occlusion of the binding site by the lid and inhibiting p53 binding to MDMX. The fact that R18 is strictly conserved (Supplementary Figure 3) indicates that it could play a key functional role, thus lending support to our proposed model.

This mechanism of disrupting the binding of p53 to MDMX upon cellular stress activation is rather similar to that in the MDM2–p53 system. The binding of p53 to MDM2 is inhibited by the phosphorylation of S17 in the MDM2 N-terminal lid [31, 32]. The phosphorylated serine interacts with the positively charged K94 at the edge of the binding site, thus stabilizing the MDM2 lid in the closed state and inhibiting p53 binding. In the case of MDMX, the positions of the interacting phosphorylated and positively charged residues are likely reversed, as our simulations suggest that pY99 at the edge of the binding site interacts with R18 in the lid to enhance lid binding to the p53-binding cleft.

Conversely, phosphorylation of Y55 is proposed to facilitate the rebinding of p53 to MDMX following stress-induced activation by promoting movement of the N-terminal lid away from the binding pocket. This is mediated by electrostatic interactions between the phosphate group of pY55 and the N-terminus of the lid.

**Figure 9:**
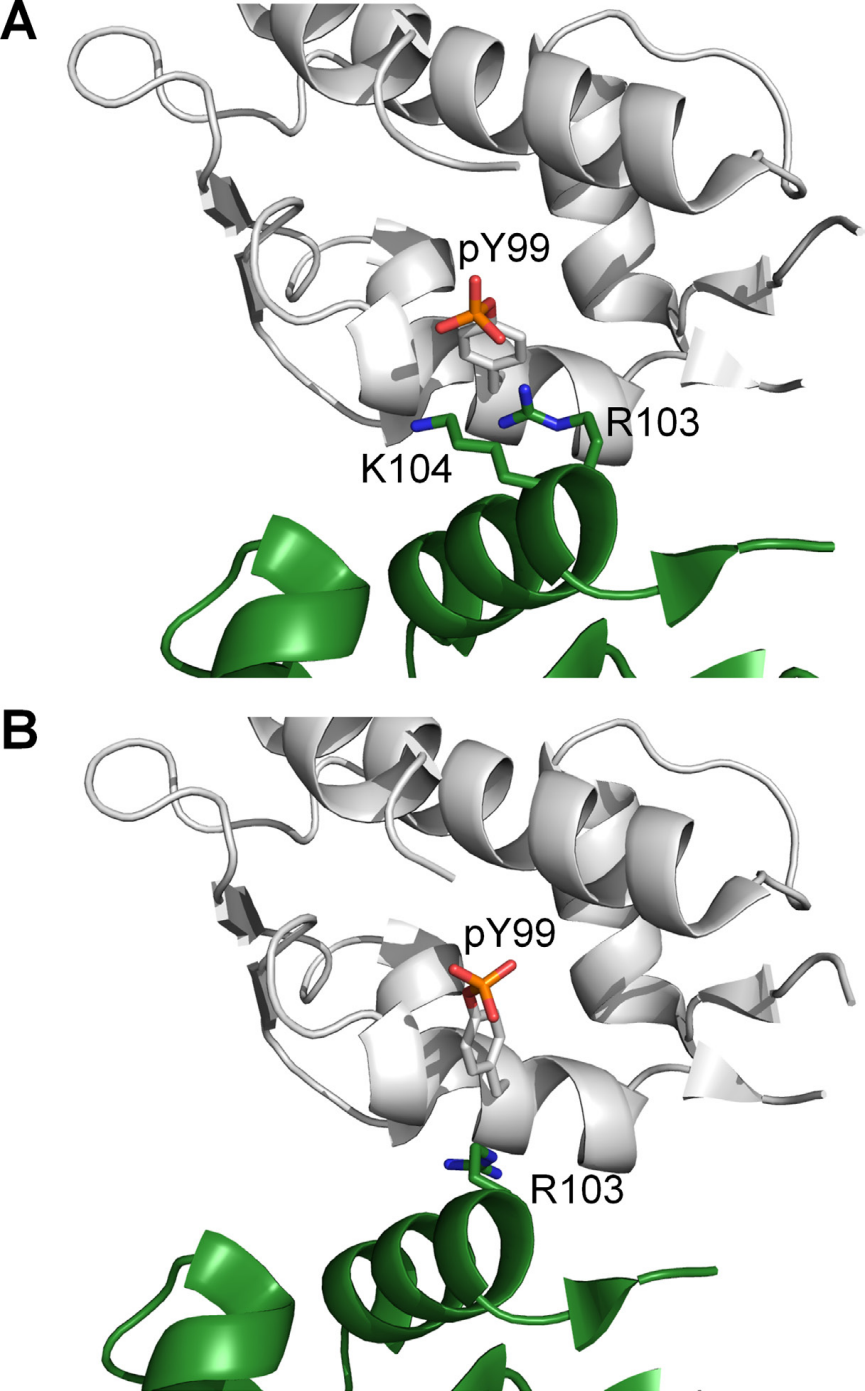
Crystal contacts with neighboring MDMX chains (green) in the vicinity of pY99 for (**A**) chain A (white) and (**B**) chain B (white) in the crystal structure of MDMX-pY99 bound to PMI (PDB 4RXZ).

We observed in our simulations that when Y99 is phosphorylated, it overwhelmingly prefers the open conformation in which it points away from the p53-binding site towards the bulk solvent. However, a recent crystal structure of MDMX-pY99 complexed with a high-affinity peptide PMI shows pY99 in the closed conformation, which contradicts our simulation observations. Closer inspection of the crystal structure revealed the effect of crystal contacts on pY99 conformation. There are two units of the MDMX–p53 complex in each asymmetric unit of the crystal structure. In one of the MDMX chains, pY99 is pinned against the p53-binding site by another MDMX chain from the neighboring asymmetric unit (Figure 9A). This crystal contact is reinforced by electrostatic interactions of the phosphate group of pY99 with the side chains of R103 and K104 from the neighboring MDMX molecule. In the other MDMX chain of the asymmetric unit, although the crystal contact is not as extensive, the presence of R103 from the neighboring MDMX molecule could be enough to prevent pY99 from adopting the open conformation (Figure 9B). These crystal contacts could also prevent R103 from forming a salt bridge with pY99, which would stabilize the latter in the open conformation. Hence, the effects of crystal packing must be considered in the interpretation of a crystal structure.

Another point of consideration is that the full-length N-terminal domain of MDMX was not used for the X-ray crystallography. The MDMX protein used for the crystallographic and biophysical binding experiments described in the study contained only residues 24–108. This means that any effects that the N-terminal lid may have on the interaction between MDMX and PMI will be missed. This could have important implications on the experiments, especially since the interaction between R18 in the lid and pY99 has been shown to be vital for promoting the closed state of the lid in our simulations.

Lastly, the crystal structure shows MDMX-pY99 bound to PMI, and not p53. PMI is a shorter, more α-helical, and more potent MDMX binder than p53. As a result of these differences, the two peptides are likely to interact differently with MDMX-pY99. The authors involved in the X-ray crystallographic study report that they were unable to obtain a structure of MDMX-pY99 in complex with p53, presumably due to its much lower binding affinity compared to PMI. Therefore, it is not clear whether pY99 also adopts the closed conformation in the presence of p53. Although the binding assays do suggest that p53 peptides of various lengths bind to MDMX with decreased affinity when Y99 is phosphorylated, the MDMX protein used lack the N-terminal lid region, which as this study shows, does appear to play a role in regulating this interaction. We would expect the deleterious effect of Y99 phosphorylation to be exacerbated if a longer MDMX with its N-terminal lid were used for the binding assays.

The importance of selected interactions between the phosphorylated tyrosine residues and lid residues in regulating the lid state was further investigated in MD simulations. To prevent R18 from forming a salt bridge with pY99, it was mutated to glutamate. The effect was striking, as the lid was repelled from pY99 and the region of the binding cleft around pY99 in all the simulations. This suggests that the R18E mutation could promote the adoption of the open state by the lid, which would result in partial recovery of p53 binding affinity by MDMX-pY99. As for pY55, its interaction with the amino group at the N-terminus of MDMX was effectively nullified by N-terminal acetylation in the simulations. This resulted in the release of the lid from pY55, which was then free to compete with p53 for association with the binding site. This suggests that N-terminal acetylation of MDMX could neutralize the inductive effect of Y55 phosphorylation on p53 binding to MDMX.

These two MDMX modifications underline the role of the lid in modulating p53 binding to phosphorylated MDMX, and would be very useful to test the validity of our proposed model. Given that our simulations show that glutamate is an unsuitable phosphomimetic for both pY99 and pY55, alternative approaches are needed to study the effect of these modifications on phosphorylated MDMX [33, 34]. One particularly useful technique is native chemical ligation, which has been used for the total chemical synthesis of phosphorylated MDMX (13). Not only does it allow for site-specific incorporation of phosphotyrosines, it could also facilitate the production of N-terminal acetylated proteins. Binding assays may then be carried out to compare the binding of p53 to the unmodified and modified phosphorylated MDMX proteins.

In conclusion, our study reveals that the N-terminal lid region of MDMX does play an important role in the regulation of p53 binding to phosphorylated MDMX. Multiple extensive MD simulations of MDMX in different phosphorylated states suggest that both Y99 and Y55 phosphorylations have a profound effect on the N-terminal lid state, which in turn affects p53 binding. Phosphorylated Y99 was observed to interact strongly with the lid residue R18 via a salt bridge. This promotes the adoption of the closed lid state, which inhibits p53 binding and results in stress-induced p53 activation. Conversely, Y55 phosphorylation allows for the formation of several hydrogen bonds and salt bridges with the N-terminal residues, which shifts the lid away from the p53-binding site and promotes the adoption of the open lid state. This acts as a negative feedback mechanism that results in the recovery of p53 binding and reduction of p53 activity. We have also demonstrated that N-terminal acetylation and mutation of R18 to Glu in MDMX can be used to test the validity of the model proposed here. Our study here suggests a key role of the N-terminal lid in MDMX function. Further investigation into its structure and function could have important implications for the development of MDMX inhibitors for cancer treatment. It has been suggested that the binding of nutlin and benzodiazepinedione-based MDM2 inhibitors are unaffected by the presence of the lid while the potency of piperidinone-based MDM2 inhibitors are improved in the presence of the lid [32]. This is because the latter compounds engage in stabilizing hydrophobic contacts with residues at the base of the lid and avoid undesirable polar contacts with the lid by interaction with core residues. A thorough study of the effect of the MDMX lid on inhibitor binding should be carried out so that small-molecule MDMX inhibitors could be optimally designed to exploit interactions with the lid region for affinity improvement. Also, the fact that PMI is able to bind to MDMX-pY99 [15] suggests that shorter or more helical peptides that are able to steer clear of pY99 upon binding could have higher cellular efficacy, as they would be agnostic to the phosphorylation state of Y99 and thus able to target both the unphosphorylated and phosphorylated forms of MDMX.

## MATERIALS AND METHODS

### Starting structure – MDMX without lid

The crystal structure of the N-terminal domain of MDMX (residues 23–109) bound to the transactivation domain of p53 (residues 17–28) was obtained from the Protein Data Bank (PDB) structure 3DAB (16). Chains A and B were used to initiate the MD simulations (set 1, Supplementary Table 1). The missing p53 residue N29 was added using PyMOL [35]. The apo form of MDMX was generated for simulation set 2 (Supplementary Table 1) by removing the p53 peptide from the 3DAB structure.

### Starting structure – MDMX with N-terminal lid

The N-terminal lid of MDMX (residues 1–22) was modeled using the N-terminal lid of apo MDM2 in the PDB structure 1Z1M (19) as the template. The N-terminal domain sequences of MDMX and MDM2 were first aligned (Figure 1) with ClustalX [36]. Using the MMTSB toolset [37], the 24 solution structures of MDM2 were then split into three clusters. The structure with the lowest root mean square deviation (RMSD) from each cluster centroid was selected. These three selected MDM2 structures were used as templates by MODELLER [38] to generate models of the MDMX N-terminal lid. The lid models with the lowest RMSD from each of the chosen template MDM2 structures were used as the starting structures for simulation sets 3–5 respectively (Supplementary Table 1).

### Rebinding of p53 peptide to apo MDMX

In simulation set 6 (Supplementary Table 1), the p53 peptide was reintroduced into MDMX trajectory structures obtained from previous simulation runs. In these cases, the structure of the p53 peptide was taken from the PDB structure 3DAB, following alignment of the MDMX crystal and trajectory structures.

### Molecular dynamics simulations

Crystallographic water molecules present in the crystal structure were retained. All protein and peptide structures were capped by acetyl and N-methyl groups at their N- and C- termini respectively, except for the N-terminus of the MDMX lid models. Residue protonation states were determined and missing hydrogen atoms added by PDB2PQR [39]. All systems were solvated with TIP3P [40] water molecules in a periodic truncated octahedron box using the LEaP program in AMBER 14 [41]. The walls of the box were at least 10 Å away from the structure. Charges were neutralized with either sodium or chloride ions using the LEaP program.

A similar protocol was used for all MD simulations, which were carried out using the PMEMD module of AMBER 14 with the ff99SB force field [42]. Parameters for phosphotyrosine were used as described by Homeyer *et al.* [43]. The SHAKE algorithm [44] was used to constrain all bonds involving hydrogen atoms to allow for a time step of 2 fs. During energy minimization and equilibration, non-hydrogen atoms were restrained by weak harmonic positional restraints with a force constant of 2 kcal mol^-1^ Å^-2^. The steepest descent algorithm was used for 500 steps, followed by another 500 steps using the conjugate gradient algorithm to minimize the energy of the system. Following this, the system was gradually heated to 300 K at constant volume for 50 ps, and then equilibrated at a constant pressure of 1 atm for 50 ps. Thereafter, the atomic restraints were removed for an unrestrained equilibration run of 2 ns, followed by a production run at 1 atm and 300 K. Temperature was kept constant with the Langevin thermostat [45] by maintaining a collision frequency of 2 ps^-1^ while the Berendsen barostat [46] maintained a constant pressure of 1 bar with pressure relaxation time of 2 ps. The lengths of the various production runs are summarized in Supplementary Table 1.

### Analysis

PyMOL [35] and Visual Molecular Dynamics (VMD) [47] were used for visualization of structures. Side chain dihedral angles (χ_1_) were calculated using the nitrogen, alpha carbon (Cα), beta carbon (Cβ) and gamma carbon (Cγ) atoms of the residue. The χ_1_ angles were used to determine the conformation of Y99/pY99 in MDMX.

Binding free energies were calculated in AMBER 14 with the Molecular Mechanics/Generalized Born Surface Area (MM/GBSA) method (18), using 200 equally spaced snapshots from the last 30–50 ns of the trajectories, depending on when equilibration was completed. This was determined with the use of RMSD plots reflecting the Cα RMSD of trajectory structures from the starting structure. The free energies of the complex (G_com_), receptor (G_rec_) and ligand (G_lig_) were evaluated as the sum of molecular mechanical energies (E_MM_), solvation energies (G_sol_) and entropy (TS). The final binding free energy change, ΔG, was obtained using the equation ΔG = G_com_ −G_rec_−G_lig_.

G = EMM + Gsol− TS

$$\Delta \text{G }={\text{ G}}_{\text{com}}-{\text{ G}}_{\text{rec}}-{\text{ G}}_{\text{lig}}$$

Molecular mechanical energies were obtained with the sander module of AMBER 14. Polar and nonpolar contributions to the solvation free energy were calculated with the pbsa program using the modified Generalized Born (GB) model described by Onufriev *et al.* [48], and the molsurf [49] program, respectively. Entropies were estimated by normal mode analysis [50] using the nmode program, based on 50 equally-spaced snapshots from the last 30–50 ns of the trajectories.

Hydrogen bond analysis was performed on the last 100 ns of the MD simulations using the hbond command in AMBER 14, which determines the hydrogen bonds in each frame of a simulation using geometric criteria. Only hydrogen bonds with a distance of less than 3.5 Å between the acceptor and donor heavy atoms, and with a bond angle of at least than 135° were considered. Only hydrogen bonds present in at least 5% of the total number of trajectory frames were considered.

## SUPPLEMENTARY MATERIALS FIGURES AND TABLE




