# Supplementary material for: Role of the N-terminal lid in regulating the interaction of phosphorylated MDMX with p53
Source: Oncotarget. 2017 Dec 1;8(68):112825–40. doi: 10.18632/oncotarget.22829 (PMC5762554; doi:10.18632/oncotarget.22829)
Supplement: Supplementary file 1 [file oncotarget-08-112825-s001.pdf]

Role of the N-terminal lid in regulating the interaction of phosphorylated MDMX with p53

SUPPLEMENTARY MATERIALS

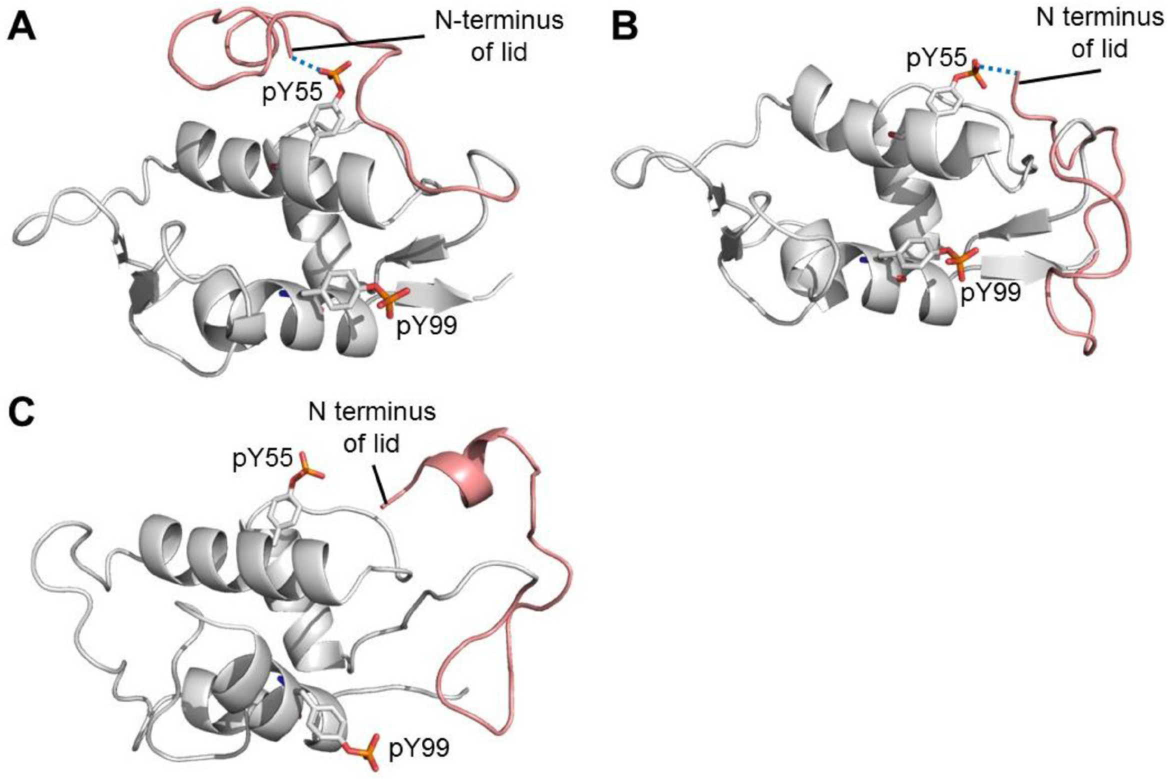

Supplementary Figure 1: Starting MDMX-pY99-pY55 structures for simulation sets 6–9, obtained from the final trajectory structures of simulations 3.3, 4.3 and 5.3. All structures are of apo MDMX-pY99-pY55 (white) with the N-terminal lid (pink) out of the binding pocket. Blue dashes represent polar interactions between pY55 and the N-terminus of the lid. (**A**) Final structure of MDMX with lid model 1 from run 3.3 replicate 2. Interactions were observed between pY55 and M1. (**B**) Final structure of MDMX with lid model 2 from run 4.3 replicate 1. Interactions were observed between pY55 and M1 and T2. (**C**) Final structure of MDMX with lid model 3 from 5.3 replicate 3. No interactions were observed between pY55 and the N-terminus of the lid.

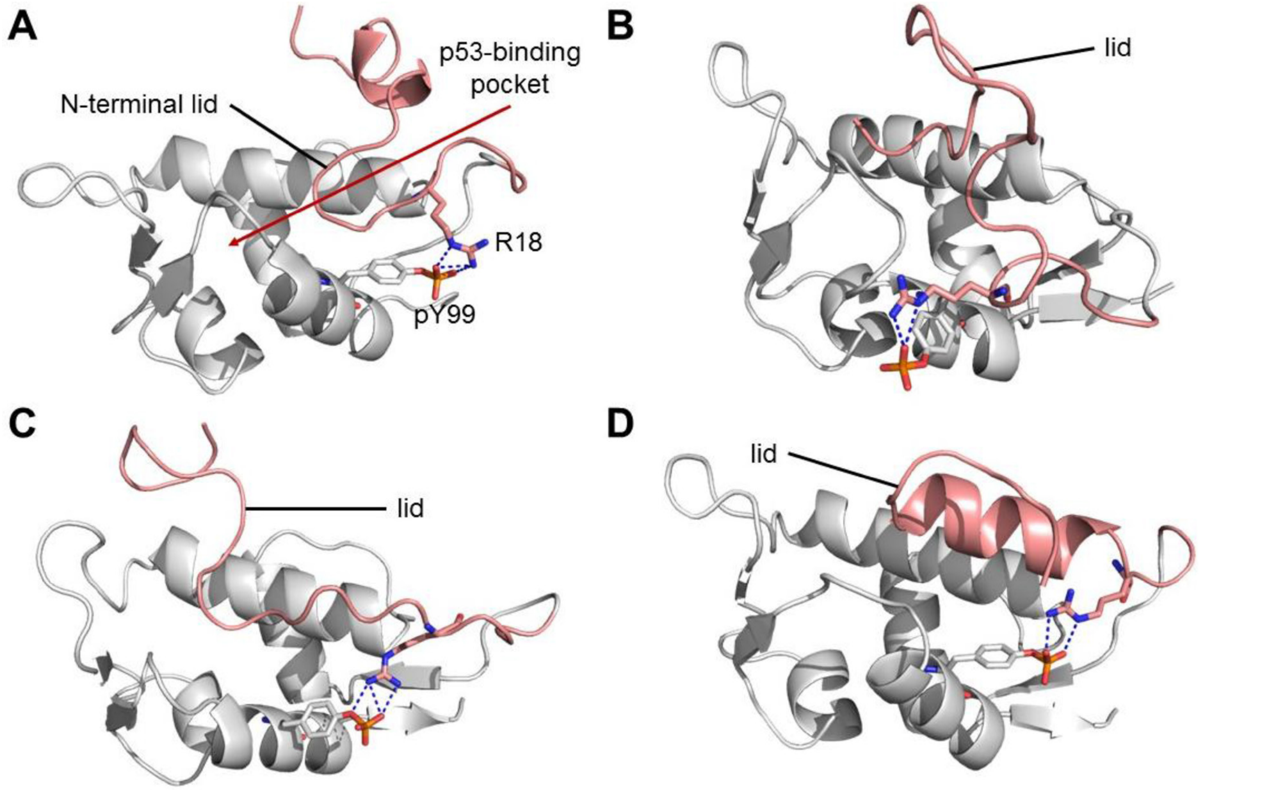

Supplementary Figure 2: Final trajectory structures of MDMX-pY99 (white) from simulation sets 3–5 with pY99-R18 interaction and the N-terminal lid (pink) in the p53-binding pocket. Interactions between pY99 and R18 are shown as blue dashes. (**A**) Final structure of run 3.2 replicate 2. (**B**) Final structure of run 3.2 replicate 3. (**C**) 200 ns structure of run 3.4 replicate 1. (**D**) 200 ns structure of run 5.4 replicate 2.

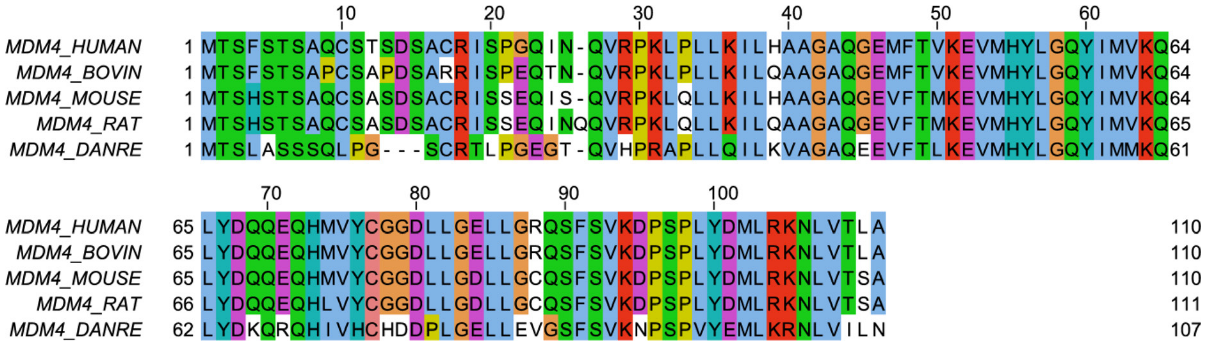

Supplementary Figure 3: Multiple sequence alignment of the N-terminal domains of MDMX from different species.

Supplementary Table 1: Summary of molecular dynamics simulation runs performed (pY99 - phosphorylated Y99; pY55 - phosphorylated Y55). See Supplementary_Table_1
