# Supplementary material for: Role of the N-terminal lid in regulating the interaction of phosphorylated MDMX with p53
Source: Oncotarget. 2017 Dec 1;8(68):112825–40. doi: 10.18632/oncotarget.22829 (PMC5762554; doi:10.18632/oncotarget.22829)
Supplement: Supplementary file 2 [file oncotarget-08-112825-s002.docx]

**Supplementary Table 1: Summary of molecular dynamics simulation runs performed.**

**(pY99 - phosphorylated Y99; pY55 - phosphorylated Y55)**

| **Set** | **Simulation no.** | **Starting structure** | **Ligand** | **MDMX modifications** | **Number of runs x simulation length (ns)** |  |
| --- | --- | --- | --- | --- | --- | --- |
| 1 | 1.1 | MDMX | p53 | none | 3 × 100 |  |
|  | 1.2 | MDMX | p53 | pY99 | 3 × 100 |  |
|  | 1.3 | MDMX | p53 | pY99, pY55 | 3 × 100 |  |
| 2 | 2.1 | MDMX | none | none | 3 × 100 |  |
|  | 2.2 | MDMX | none | pY99 | 3 × 100 |  |
|  | 2.3 | MDMX | none | pY99, pY55 | 3 × 100 |  |
| 3 | 3.1 | Lid model 1 | none | none | 3 × 300 |  |
|  | 3.2 | Lid model 1 | none | pY99 | 3 × 300 |  |
|  | 3.3 | Lid model 1 | none | pY99, pY55 | 3 × 300 |  |
|  | 3.4 | Lid model 1 | none | none at 0 ns, pY99 at 100 ns, pY55 at 200 ns | 3 × 300 |  |
| 4 | 4.1 | Lid model 2 | none | none | 3 × 300 |  |
|  | 4.2 | Lid model 2 | none | pY99 | 3 × 300 |  |
|  | 4.3 | Lid model 2 | none | pY99, pY55 | 3 × 300 |  |
|  | 4.4 | Lid model 2 | none | none at 0 ns, pY99 at 100 ns, pY55 at 200 ns | 3 × 300 |  |
| 5 | 5.1 | Lid model 3 | none | none | 3 × 300 |  |
|  | 5.2 | Lid model 3 | none | pY99 | 3 × 300 |  |
|  | 5.3 | Lid model 3 | none | pY99, pY55 | 3 × 300 |  |
|  | 5.4 | Lid model 3 | none | none at 0 ns, pY99 at 100 ns, pY55 at 200 ns | 3 × 300 |  |
| 6 | 6.1 | Final structure of 3.3 replicate 2 | p53 | pY99, pY55 | 1 × 300 |  |
|  | 6.2 | Final structure of 4.3 replicate 1 | p53 | pY99, pY55 | 1 × 300 |  |
|  | 6.3 | Final structure of 5.3 replicate 3 | p53 | pY99, pY55 | 1 × 300 |  |
| 7 | 7.1 | Final structure of 3.3 replicate 2 | none | N-terminal acetylation | 3 × 300 |  |
|  | 7.2 | Final structure of 3.3 replicate 2 | none | M1E | 3 × 300 |  |
|  | 7.3 | Final structure of 3.3 replicate 2 | none | T2E, S3E | 3 × 300 |  |
|  |  |  |  |  |  |  |

| **Set** | **Simulation no.** | **Starting structure** | **Ligand** | **MDMX modifications** | **Number of runs x simulation length (ns)** |
| --- | --- | --- | --- | --- | --- |
| 8 | 8.1 | Final structure of 4.3 replicate 1 | none | N-terminal acetylation | 3 × 300 |
|  | 8.2 | Final structure of 4.3 replicate 1 | none | M1E | 3 × 300 |
|  | 8.3 | Final structure of 4.3 replicate 1 | none | T2E, S3E | 3 × 300 |
| 9 | 9.1 | Final structure of 5.3 replicate 3 | none | N-terminal acetylation | 3 × 300 |
|  | 9.2 | Final structure of 5.3 replicate 3 | none | M1E | 3 × 300 |
|  | 9.3 | Final structure of 5.3 replicate 3 | none | T2E, S3E | 3 × 300 |
| 10 | 10.1 | Final structure of 3.2 replicate 2 | none | R18E | 1 × 300 |
|  | 10.2 | Final structure of 3.2 replicate 3 | none | R18E | 1 × 300 |
|  | 10.3 | 200 ns structure of 3.4 replicate 1 | none | R18E | 1 × 300 |
|  | 10.4 | 200 ns structure of 5.4 replicate 2 | none | R18E | 1 × 300 |
| 11 | 11.1 | Lid model 1 | none | Y99E | 3 × 300 |
|  | 11.2 | Lid model 1 | none | Y99E, Y55E | 3 × 300 |
| 12 | 12.1 | Lid model 2 | none | Y99E | 3 × 300 |
|  | 12.2 | Lid model 2 | none | Y99E, Y55E | 3 × 300 |
| 13 | 13.1 | Lid model 3 | none | Y99E | 3 × 300 |
|  | 13.2 | Lid model 3 | none | Y99E, Y55E | 3 × 300 |
